# Predicting facility delivery and its determinants among reproductive-age women in Ethiopia using machine learning algorithm: evidence from performance monitoring for action Ethiopia 2019 dataset

**DOI:** 10.1186/s12884-026-09354-0

**Published:** 2026-05-30

**Authors:** Siraj Muhidin Degefa, Beriso Alemu Hailu, Naol Gonfa Serbessa, Asmamaw Ketemaw Tsehay, Tigist Tollessa Sedi, Eden Ketema Woldekidan, Mohammedjud Hassen Ahmed, Jibril Bashir Adem, Agmasie Damtew Wale, Habtamu Alganeh Guadie, Mulusew Andualem Asemahagn

**Affiliations:** 1 Department of Health Informatics, College of Health Science, Mattu University, Mettu, Ethiopia; 2 School of Public Health, College of Medicine and Health Sciences, Bahir Dar University, Bahir Dar, Ethiopia; 3 Department of Health Informatics, College of Medicine and Health Sciences, Arbaminch University, Arbaminch, Ethiopia; 4 Department of Health Informatics, School of Public Health, Asrat Woldeyes Health Science Campus, Debre Berhan University, Debre Berhan, Ethiopia; 5 Department of Public Health, College of medicine and Health sciences, Arsi University, Asella, Ethiopia

**Keywords:** Facility delivery, Women, Determinants, Ethiopia

## Abstract

**Background:**

Facility delivery attended by skilled health professionals is a critical strategy for reducing maternal and neonatal mortality. Despite substantial efforts to expand maternal health services in Ethiopia, a large proportion of births still occur at home. This study aimed to predict facility delivery and identify its associated factors among reproductive-age women in Ethiopia using machine learning approaches.

**Methods:**

This study used secondary data from the 2019 Performance Monitoring for Action (PMA) Ethiopia cross-sectional household and female survey. A weighted sample of 5,413 women aged 15–49 years who had a recent birth was included in the analysis. Data extraction and preprocessing were conducted using STATA version 17, while machine learning models were implemented using Python version 3.11.5. Seven supervised machine learning algorithms were developed to predict facility delivery. Model performance was evaluated using accuracy, precision, recall, F1 score, and the area under the receiver operating characteristic curve (AUC). Data preparation included feature engineering, data splitting, handling missing values, addressing class imbalance, and outlier detection. SHapley Additive exPlanations (SHAP) were applied to interpret the contribution of important predictors to model predictions.

**Results:**

Among the evaluated models, the Random Forest algorithm demonstrated the best predictive performance, achieving 74.15% accuracy, 72.58% recall, 76.27% F1-score, and 80.36% precision on test data. The most influential predictors of facility delivery included higher household wealth status, secondary or higher maternal education, television ownership, discussion of delivery location with a partner, partner encouragement for antenatal care attendance, family planning use, maternal age between 20 and 34 years, age at first sexual intercourse of 19 years or older, awareness of emergency contacts, and completion of primary education.

**Conclusions:**

Interpretable machine learning approaches can provide useful insights for identifying factors associated with facility delivery. The findings highlight key socioeconomic and behavioral characteristics that may inform targeted strategies to improve utilization of facility-based delivery services and support efforts to enhance maternal health outcomes in Ethiopia.

## Introduction

Health facility delivery refers to childbirth that occurs in government, public, or private health institutions such as hospitals or clinics [1]. It plays a critical role in improving maternal and neonatal health outcomes by ensuring access to skilled birth attendants and timely medical care during labor and delivery [2, 3]. The location of delivery substantially influences the health of both the mother and the newborn, particularly during the postpartum period, which is a vulnerable time for complications [4]. The World Health Organization recommends that all women have access to essential maternal health services, including skilled birth attendance, antenatal care, and postnatal follow-up [5]. Despite these recommendations, home births remain common in many low- and middle-income countries, increasing the risk of preventable complications such as prolonged labor, retained placenta, postpartum hemorrhage, and maternal infections [6, 7].

Globally, more than 200 million conceptions occur annually [8], yet 40% of these pregnancies result in complications for women [9]. In 2020, almost 800 women died daily from preventable causes related to pregnancy and childbirth, equating to one woman every two minutes [10]. Reducing the global maternal mortality ratio (MMR) to fewer than 70 deaths per 100,000 births is one of the Sustainable Development Goals (SDGs) [11–13]. Research indicates that non-institutional deliveries frequently lead to maternal fatalities in low-resource settings like Ethiopia [14]. Contributing factors include poor socioeconomic status, limited access to obstetric facilities, inefficient emergency referral mechanisms, and long travel distances for rural women [15–19]. Unsafe delivery practices are responsible for over 50% of maternal mortality in underdeveloped countries [20].

Despite significant progress over the past two decades, the global number of maternal deaths remains unacceptably high, with approximately 295,000 women dying in 2017 from pregnancy and childbirth-related causes [21]. In developing nations, over half of births occur outside medical facilities without a trained birth attendant present [22]. Maternal deaths in Southern Asia and Sub-Saharan Africa account for 86% of all maternal fatalities worldwide [21]. Nonetheless, evidence shows that in countries where medical professionals handle over 80% of deliveries, the Maternal Mortality Rate (MMR) is less than 200 per 100,000 live births [23]. In 2017, about two-thirds (196,000) of maternal deaths occurred in Sub-Saharan Africa, and one-fifth (58,000) occurred in Southern Asia [24].

In Ethiopia, despite government efforts to expand healthcare facilities and promote institutional delivery services, only 25% of women give birth in medical institutions [25]. This low rate of facility delivery has significant implications for maternal mortality. Based on a World Bank report, the trend of Ethiopia’s maternal mortality rate reveals that in 2020, it was 267.00, 9.18% lower than in 2019, 294.00, 5.77% lower than in 2018, 312.00, 10.34% lower than in 2017, and 348.00, 2.25% lower than in 2016 [10].

Over the years, numerous researches have looked for characteristics related to the place of delivery in different parts of Ethiopia [15, 18, 26–30]. These studies have consistently reported substantial connections with maternal education, household wealth, age, distance from a health institution, or lack of transportation [31]. Despite these findings, the studies primarily use conventional statistical methods and do not provide insights beyond what is already known. The limitations of earlier research, such as small sample sizes or limited geographic coverage, hamper generalizability. Large-scale datasets, such as PMA 2019, can offer a more thorough insight into Ethiopian facility delivery. Ethiopian studies on facility delivery drivers primarily use conventional statistical methods, leaving a gap in the literature for machine learning algorithms to forecast facility delivery.

Therefore, this study aimed to predict facility delivery and its determinants among reproductive-age women in Ethiopia using machine learning methods. This approach revealed complex patterns and linkages in the data, offering a more nuanced perspective of the factors impacting facility delivery. The research utilized a comprehensive analysis considering sociodemographic, socioeconomic, and reproductive (obstetric) history factors. By utilizing a sizable dataset from PMA 2019, this study employed supervised machine learning to predict facility delivery and its determinants, providing insights for evidence-based practices and policy enhancement to improve Ethiopia’s health outcome.

## Method

### Study design, setting and period

A Population-Based Cross-sectional Household and Female survey study design was conducted, PMA2019 dataset for the year 2019 in Ethiopia. Ethiopia is a Federal Democratic Republic made up of two chartered administrative cities (Addis Ababa City administration and Dire Dawa city council) and 9 National Regional states (Afar, Amhara, Oromia, Somali, Benishangul-Gumuz, Gambella, Tigray, Southern Nations Nationalities and People Region (SNNPR), and Harari). The two city administrative councils, together with the national regional states, are further subdivided into around 15,000 kebeles and 800 woredas (districts) [32]. With a population estimated at 118 million in 2021, Ethiopia occupies 1,119,683 square kilometers, and more than 84% of them reside in rural areas. Yet again More than 353 public hospitals, 3,706 public health centers, and 17,561 health posts are part of the nation’s three-tiered healthcare delivery system. This cross-sectional survey was carried out from September 2019 to December 2019.

### Source population

All reproductive-age [15–49] Ethiopian women were sources population to this study.

### Study population

All reproductive-age [15–49] Ethiopian women in the enumeration areas were study population to this study.

### Inclusion and exclusion criteria

#### Exclusion criteria

For this analysis, missing or incomplete data in the PMAET 2019 Dataset were excluded.

#### Inclusion criteria

All reproductive-age Ethiopian women [15–49] who reported being pregnant or ever gave birth including in the past six weeks before the survey were included.

### Sample size determination and sampling methods

Weighted samples of 5,413 women who were of reproductive age were included in this analysis. The weighting of the sample size in this analysis was account for changes in the probability of selection and non-response.

The PMA Ethiopia 2019 Cross-sectional Household and Female (HQFQ) survey employed a two-stage cluster design with main regions and urban-rural areas serving as strata. For the regions of Tigray, Amhara, Oromia, and SNNP, EAs were drawn separately from urban and rural strata within the region. For the regions of Afar, Somali, Benishangul-Gumuz (BG), Gambella, and Harari, the EAs were randomly selected with probability-proportional to-size from within the region, without urban/rural stratification. Addis is exclusively urban and thus all EAs were drawn from within the region without stratification. In total 265 enumeration areas (EAs) were chosen from the Central Statistics Agency’s master sample frame. Within each enumeration region, a cross-section of 35 households was chosen at random. The cross-sectional survey was open to all females in the chosen households between the ages of 15 and 49. The cross-sectional survey was finished by 8,837 women and 9,108 homes [32].

### Study variables

#### Dependent variable

The outcome variable was taken as binary response women gave birth at home and others home, coded as “0=no” which is home delivery, and women gave birth in different governmental health facilities, private health facilities, and non-governmental health facilities coded as “1=yes” which is health facility delivery

#### Independent variables

Independent variables were selected based on prior literature and data availability in the PMA Ethiopia 2019 dataset. Sociodemographic and economic variables included place of residence (urban/rural), maternal age (15–19, 20–34, 35–49 years), maternal birthplace (urban/rural), religion, region, marital status, educational level (no education, primary, secondary or higher), household wealth quintile, and media access (ownership of radio or television).

Reproductive health and family planning–related variables included age at first sexual intercourse, ever use of family planning methods (yes/no), number of antenatal care (ANC) visits (categorized as none, 1–3, ≥ 4), partner encouragement for ANC (yes/no), unmet need for family planning (yes/no), pregnancy intention (intended/unintended), desire for children before the index pregnancy (yes/no), discussion with partner about place of delivery (yes/no), experience of forced pregnancy (yes/no), preferred birth interval (< 24 months / ≥24 months), parity (nulliparous, 1–2, ≥ 3), and whether the partner had another wife (yes/no).

Prior to model training, categorical variables were encoded using appropriate numerical representations to enable machine learning analysis. Following preprocessing, dimensionality reduction was performed using recursive feature elimination with cross-validation (RFECV) to retain the most informative predictors. Only variables selected through RFECV were included in the final model training and evaluation.

### Operational and term definition

#### Facility delivery

Facility delivery refers to childbirth that takes place in a medical facility equipped with resources and staffed by qualified medical personnel capable of identifying, treating, or referring obstetric issues [33], This includes any delivery in a healthcare setting where trained birth attendants are present, as determined by a series of yes/no questions [34].

#### Wealth quantile

Wealth quintile is a measure used to categorize individuals or households into different socioeconomic groups based on their relative wealth or economic status. It divides the population into five equal parts, each representing 20% of the population, ranked from the poorest to the wealthiest [35].

#### Access to media devices

Access to media devices refers to the ownership or availability of electronic devices that enable individuals to consume various forms of media content [36]. Owning a television, radio or both considered as access to media device.

### Data source/collection

The dataset utilized in this study was obtained through a formal request made after registering and subscribing to the PMA Survey on their website, https://www.pmadata.org. PMA-Ethiopia is a five-year (2019–2023) project in collaboration with Addis Ababa University, Johns Hopkins University, and the Federal Ministry of Health. It consists of three distinct study activities: yearly cross-sectional surveys of women aged 15–49; longitudinal surveys of women who are pregnant or have given birth; and yearly service delivery point surveys of health facilities [32]. For this analysis we used yearly cross-sectional survey of women aged 15–49 which was done in 2019, and weighted sample of 5,413 women of reproductive age was acquired, and then proper data preparation was undertaken to make the data suitable for the ML task.

### Data processing and analysis

A supervised machine learning (ML) technique was applied using secondary data from the PMA Ethiopia 2019 Cross-sectional Household and Female survey. In order to forecast facility delivery, this study was employed the basic framework found in existing research that is based on Yufeng Guo’s 7 Steps of Machine Learning. The framework delineates the seven stages involved in supervised machine learning, namely: data gathering, data preprocessing, model selection, model training, model assessment, parameter adjustment, and forecasting [37, 38]. For variable extraction, the statistical software STATA version 17 was utilized, Using Jupyter Notebook, the scikit-learn and XGBoost packages, machine learning (ML) methods was built in Python 3.11.5. To guarantee national representativeness, the PMA Ethiopia 2019 survey uses a multistage cluster sampling design and offers sampling weights. In order to account for uneven probabilities of selection and non-response, the female survey sample weights included in the dataset were used in this study’s descriptive analysis using STATA version 17. In order to preserve the study population’s representativeness, the machine learning analysis used the weighted dataset.

#### Data preparation (pre-processing)

Data cleaning, feature engineering, dimensionality reduction, data splitting and handling class imbalances were all preprocessing tasks that we had completed. Following the extraction of the data, the data was cleaned, which includes handling missing values and unbalanced result variable categories in addition to locating and eliminating outliers from the dataset.

#### Data cleaning

After the data is retrieved, the first phase is called “data cleaning,” which includes handling missing values, identifying and eliminating outliers, and addressing imbalanced outcome variable categories in the dataset. In this study, missing values in the independent variables were imputed using the k-nearest neighbors (KNN) imputation method implemented in the Python *scikit-learn* library, with the number of neighbors set to k = 5. KNN imputation was used since it is a more reliable and sensitive method for missing value imputation [39].

#### Feature engineering

In order to improve model accuracy on unobserved data, raw data’s were transformed into features that more correctly reflect the underlying problem for predictive models [40]. Thus, one-hot-encoding techniques were employed using the Pandas ‘get_dummies’ method to encode categories to dummy variables, with each category as a separate variable coded as 0 or 1 to indicate presence and absence, respectively [41]. In machine learning, one-hot encoding is a popular feature engineering method for handling categorical data. To enable the machine learning algorithm to treat each category as a separate entity, it entails generating a new binary (0 or 1) feature for each unique category in the original categorical variable [42].

#### Dimensionality reduction

Through the process of dimensionality reduction, the input variable count for the predictive model was reduced using Recursive Feature Elimination with Cross-Validation (RFECV). This technique works by recursively removing the least important features and re-training the model, leading to a simpler predictive model that may perform better when making predictions on new data [43]. The analysis indicated that fewer input variables contributed to improved model generalization.

#### Data splitting

The conventional 80/20 split technique was used in this study, allocating 20% of the data for testing the model and 80% for training. Given the limited number of examples, the study employed tenfold cross-validation to ensure efficient use of the data during model training [44]. K-Fold divides all observations into folds, or equal-sized sample groups, and k-1 folds in order to train the prediction function. On the excluded fold, k tests were performed one after the other [37]. The k-fold cross-validation performance statistic was the average of the findings calculated during the loop. Then Imbalanced Categories of the training set was handled using SMOTE (Synthetic Minority Over-sampling Technique) to balance the class distribution. 

#### Handling class imbalances

To address imbalance in the outcome variable, the Synthetic Minority Oversampling Technique (SMOTE) was applied during model training. The outcome variable, health facility delivery, showed a moderate class imbalance in the dataset. Of the total 5,413 observations, 2,313 (42.73%) represented non–facility delivery, while 3,100 (57.27%) represented facility delivery.

Although the imbalance was not extreme, it had the potential to bias model learning toward the majority class, which could reduce predictive sensitivity for the minority category. To minimize this risk and improve model robustness, SMOTE was used to generate synthetic samples for the minority class within the training dataset only. This approach helped balance class representation, enhance model learning, and improve the generalizability of predictions related to facility delivery outcomes.

#### Model selection

After data preparation and splitting into training and testing sets, appropriate models were selected for model training. Because the outcome variable health facility delivery has two mutually exclusive categories (yes or no), the prediction task was formulated as a binary classification problem. Accordingly, classifiers suitable for categorical outcomes were applied. To identify the most effective algorithm for predicting factors associated with health facility delivery among reproductive-age women in Ethiopia, seven widely used machine learning algorithms were evaluated: logistic regression (LR), Random Forest (RF), K-nearest neighbors (KNN), Extreme Gradient Boosting (XGBoost), adaptive boosting (AdaBoost), Naïve Bayes (NB), and Support Vector Machines (SVM). These algorithms were deliberately selected to represent a broad range of modeling strategies, including linear models, ensemble learning methods, probabilistic classifiers, and instance-based approaches [37]. This comprehensive comparison allowed for the identification of the most suitable algorithm based on predictive performance on the study dataset.

#### Model training

Tenfold cross-validation was used to compare the performance of the chosen classifiers, who were then trained on prepared data following the model selection process. In order to produce the final prediction on fictitious test data, the best predictive model was selected after comparison and trained using balanced training data [37].

#### Model evaluation

We determined the model’s effectiveness after training by evaluating its performance on previously unseen data that was reserved for this reason during data splitting. A common method for assessing a classification model’s efficacy was the confusion matrix, which was essentially a simple cross-tabulation of the result variable’s actual and anticipated categories [45]. Confusion metrics were utilized to compute performance metrics including overall accuracy, precision, recall, and F1 score, which were used in this investigation to assess the efficacy of the chosen classifiers. Additionally, the receiver operating characteristic (ROC) curve and its value were used to evaluate the performance of the machine learning models. An area under the ROC curve (AUC) value of 0.5 indicates no discriminative ability, equivalent to random guessing. AUC values between 0.5 and 0.7 suggest poor discrimination. AUC values between 0.7 and 0.8 are considered acceptable, while AUC values between 0.8 and 0.9 indicate good discrimination. An AUC value above 0.9 represents excellent discrimination [46].

#### Hyper parameter tuning

Hyperparameters are configuration settings that control the learning process of machine learning models, such as the learning rate, kernel parameters, and model complexity. Appropriate hyperparameter tuning is essential to optimize model performance and generalization. In this study, Optuna-based Bayesian optimization was employed for hyperparameter tuning of the selected machine learning models. Optuna efficiently explores the hyperparameter search space by iteratively evaluating parameter combinations and selecting those that maximize model performance based on cross-validation results [47]. This approach was chosen for its computational efficiency and ability to identify optimal parameter configurations without exhaustively evaluating all possible combinations.

#### Making prediction

This final step in the machine learning approach incorporated all the previously described tasks. Prediction involved estimating the outcome variable using a framework of independent variables. In this context, the key factors identified throughout the process were utilized to determine health facility delivery. The best-performing classifier, which had been identified, based on its accuracy, precision, recall, AUC, and F1 score, was then employed to predict the determinants of health facility delivery based on various characteristics.

#### Shapley additive explanations (SHAP) for model interpretation and explanation

A machine learning framework called Shapley Additive Explanations (SHAP) was used to interpret and explain models. Based on the idea of Shapley values from cooperative game theory, it offers a cohesive method for comprehending how crucial input qualities are for forecasting [48]. The goal of SHAP values is to quantify each feature’s contribution to the prediction that a machine learning model makes. When paired with various subsets of other features, they give an indication of how far each feature value deviates from its average contribution to the predictions [49]. Through the use of SHAP values, we could learn more about the ways in which certain features influenced a machine learning model’s predictions. Gaining confidence in the model’s decision-making process, recognizing significant features, identifying biases, and comprehending model behavior could all be aided by this [50].

#### Association rule mining

Association rule mining is a data-mining technique used to uncover meaningful relationships and co-occurrence patterns among variables within a dataset. An association rule consists of two components: an antecedent (IF) and a consequent (THEN), which together describe how the presence of certain attributes is related to a specific outcome.

In this study, after developing and evaluating the predictive machine-learning models, the key independent variables were further explored to better understand how different categorical factors jointly relate to health facility delivery. While the predictive models identified statistically important predictors, they did not explicitly reveal combinations of variables that tend to occur together in relation to the outcome. To address this gap, association rule mining was applied using the Apriori algorithm to identify strong IF–THEN relationships among the independent variables and facility delivery status. This approach provides additional insight into patterns of co-occurring factors that may inform targeted maternal health interventions.

##### Presentation

**Fig. 1:**
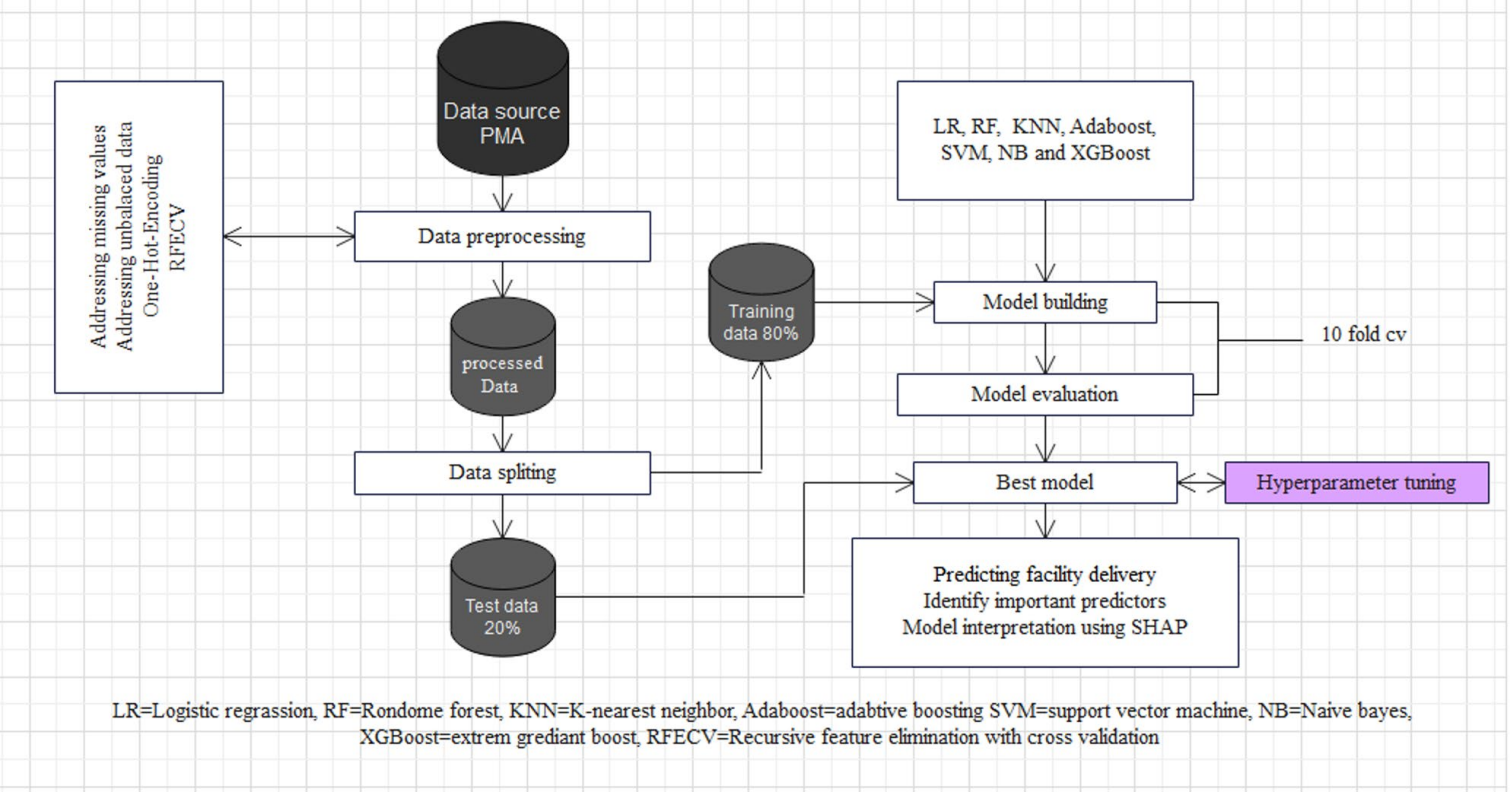
Entire data preparation and analysis plan flow chart

A combination of tables, descriptions, and figures were used to present the findings of this research. Fig. 1 shows the entire data preparation and analysis plan. 

### Data quality assurance

The dataset, received through a formal request from the PMA Survey, was collected by prestigious institutions such as Addis Ababa University, Johns Hopkins University, and the Federal Ministry of Health, which added legitimacy. A comprehensive cross-sectional survey collected extensive health and demographic data. During data cleaning, we addressed missing data, encoded categorical variables, normalized numerical features, and handled outliers. The Synthetic Minority Over-sampling Technique (SMOTE) addressed class disparities. Data consistency checks and tenfold cross-validation guaranteed integrity and robustness. The thorough documenting of data preparation processes enabled transparency and reproducibility.

### Ethical approval and consent to participate

The original Performance Monitoring for Action (PMA) Ethiopia 2019 survey received ethical approval from the Johns Hopkins Bloomberg School of Public Health Institutional Review Board and the Ethiopian Public Health Institute Ethical Review Board. All procedures involving human participants were conducted in accordance with the Declaration of Helsinki and relevant ethical guidelines. Written informed consent was obtained from all participants prior to data collection.

For this study, secondary anonymized data were obtained from the PMA data repository (https://www.pmadata.org/data/available-datasets), and permission to use the dataset was granted through the official data access process. Ethical clearance for this secondary data analysis was obtained from the Institutional Review Board of Bahir Dar University, College of Medicine and Health Sciences. As the dataset contained no identifiable personal information and no direct participant contact was involved, the requirement for informed consent was waived.

## Result

### Characteristics of participants

#### Socio-demographic and economic characteristics 

Nearly three-fourths (63.46%) of the women were a rural residents, and the mean age of the respondents was 24 (SD ± 7.8). among the total study participants (86.51%) of them were married or living with their partners, Significant number of participants were from the lowest and the highest wealth quantile accounting about (36.54%) and (45.63%) respectively, of them 46.57% were never attended formal education, Over two-thirds of respondents (70.63%) and more than three-fourths (72.22%) reported having no access to media devices such as radio and television, respectively (Table 1).

**Table 1:** Socio-demographic and economic characteristics of respondents for predicting facility delivery and its determinants among reproductive-age women in Ethiopia (PMAET 2019, *n* = 5,413)

Variable		Categories	Weighted freq.	percent
Residence		Urban	1,978	36.54
		Rural	3,435	63.46
Mothers age		15–19	189	3.49
		20–34	3,102	57.31
		35–49	2,122	39.20
Region		Tigray	699	12.91
		Afar	236	4.36
		Amhara	1,014	18.73
		Oromia	1,120	20.69
		Somali	130	2.40
		Benishangul-Gumuz	151	2.79
		SNNPR	1,049	19.38
		Gambela	244	4.51
		Harari	206	3.81
		Addis	337	6.69
		Dire Dawa	187	3.45
Marital states		Married/Living with partner	4,683	86.51
		Divorced/separated/widowed	667	12.32
		Never married	63	1.16
Birth place of mother		Urban	792	14.63
		Rural	4,621	85.37
Level of education		Never attended	2,521	46.57
		Primary	1,819	33.60
		secondary and above	1,073	19.82
Religion		Orthodox	2,528	46.70
		Protestant	1,167	21.56
		Moslem/Muslim	1,598	29.52
		Others^*^	120	2.22
Access to media device	Radio	No	3,823	70.63
		Yes	1,590	29.37
	Tv	No	3,909	72.22
		Yes	1,504	27.78
Wealth quantile		Lowest quintile	1,978	36.54
		Middle quintile	965	17.83
		Highest quintile	2,470	45.63

Note: others* indicates: catholic, Wakefata, atheist and traditional

#### Reproductive health and family planning service characteristics

A significant number 3,955 (73.06%) of women started sexual intercourse at the age of 18 years or below. Over two thirds 3,720 (68.72%) of respondents used some form of family planning methods, and 4,442 (82.06%) women reported having no unmet need for family planning followed by 4,008(74.04%) women reported having pregnancy intentions, Approximately 4,289(79.42%) women had a desire for children before becoming pregnant, the majority 4,797(88.62%) of women responded that their partners had no other wives (Table 2).

**Table 2:** Reproductive health and family planning service characteristics of respondents for predicting facility delivery and its determinants among reproductive-age women in Ethiopia (PMAET 2019, *n* = 5,413)

Variable	Categories	Weighted freq.	Percent (%)
age at first sex	18 or below	3,955	73.06
	19+	1,458	26.94
ever used family planning (FP) methods	No	1,693	31.28
	Yes	3,720	68.72
unmet need for FP	No	4,442	82.06
	Yes	971	17.94
pregnancy intention	intended to get pregnant	4,008	74.04
	intentions kept changing	225	4.16
	did not intend to get pregnant	1,180	21.80
desire for children before becoming pregnant	wanted to have a baby	4,289	79.42
	had mixed feelings about having	309	5.71
	did not want to have a baby	815	15.06
discussion with partner about place of delivery	No	2,233	41.25
	Yes	3,180	58.75
forced pregnancy	No	5,366	99.13
	Yes	47	0.87
preferred birth interval	Months	101	1.87
	Years	4,036	74.56
	Soon / Now	1,276	23.57
Parity	Nulliparous	856	13.17
	Prim parous	1,171	18.02
	Multiparous	3,474	68.82
partners have another wife	No	4,797	88.62
	Yes	616	11.38
Number of ANC visit	3 or below	5.073	93.74
	4 or above	339	6.26

### Machine learning analysis results

**Fig. 2:**
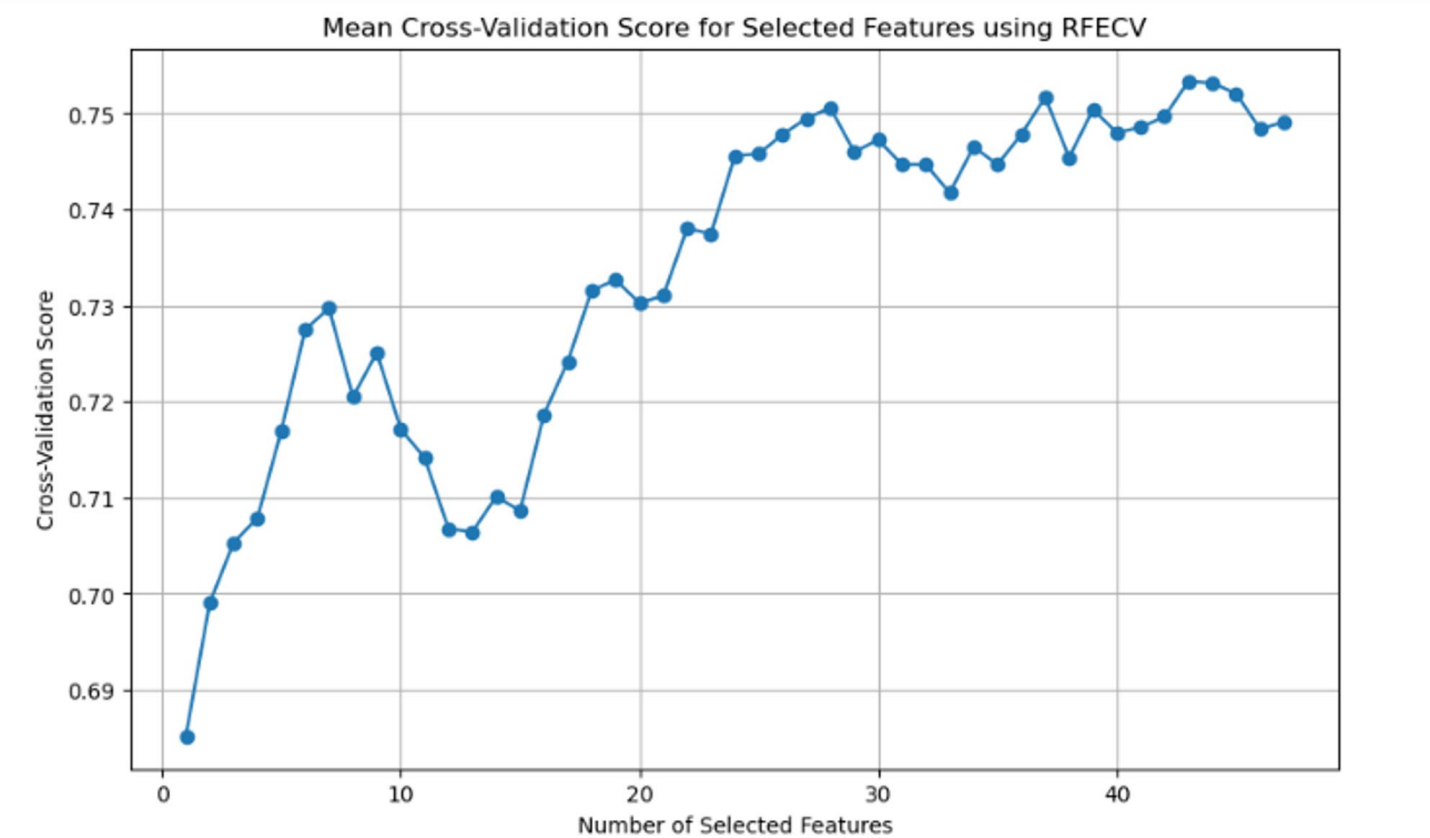
Feature selection using RFECV for predicting facility delivery and its determinants among reproductive-age women in Ethiopia (PMAET 2019, *n* = 5,413)

In this study, feature selection was performed using the Recursive Feature Elimination with 10-fold cross-validation (RFECV) technique, a wrapper method that recursively removes the least important features and re-trains the model until the desired number of features is reached, as shown in Fig. 2. The graph indicates that from the total of 51 features, cross-validation score peaks around 35 to 47 features, suggesting that this range is optimal for this particular dataset and model.

### Balancing data and model performance comparison

To address class imbalance between facility and non-facility delivery outcomes, the Synthetic Minority Over-sampling Technique (SMOTE) was applied during model training. Following data cleaning, the minority class was underrepresented relative to the majority class. SMOTE was used to generate synthetic observations for the minority class based on k-nearest neighbors (k = 5, default setting), interpolating new samples in feature space rather than duplicating existing observations. SMOTE oversampling provided 630 additional synthetic observations from the minority category of individuals who did not deliver in a health facility after dividing the data set into (80%) training and (20%) test sets. This was done to balance the outcome variable’s unequal distribution. For each class, the total facility delivery status distribution was rearranged from 1850 home delivery and 2480 facility delivery to 2480 in order to produce symmetric distributions for both categories and facilitate the creation of trustworthy prediction models.

A stratified 10-fold cross-validation was used to evaluate the models’ ability to predict facility delivery among women of reproductive age. The models’ mean accuracy, mean precision, mean area under the curve score, recall, and f1_score were all given special attention. Classifiers on the unbalanced training data were evaluated using stratified 10-fold cross-validation. The support vector machine performed best, with accuracy of 76.65%, 84.58% areas under the ROC curve, 80.20% precision, 78.71% recall, and 79.42% F1_score.

This finding may not be accurate due to the outcome variable’s unbalanced class nature, which could tilt the model in favor of the majority class. An ML model comparison was carried out following the use of the SMOTE oversampling technique to balance the training data in order to avoid the construction of a biased model. Thus, Random Forest emerged as the best accurate prediction model to forecast facility delivery among reproductive age women in Ethiopia, with 78.35% of accuracy, 86.89% areas under the ROC curve, 82.86% precision, 75.36% recall, and 77.93%F1_score. The performance of the models on balanced and unbalanced data is shown in (Table 3).

**Table 3:** Performance comparison of machine learning models for predicting health facility delivery through 10_fold cross validation on training data (PMAET 2019, *n* = 5,413)

Models	Data status	Performance (%)				
		Accuracy	AUC	F1_score	Precision	Recall
Logistic regression	Unbalanced	76.65	84.66	79.55	78.55	79.40
	Balanced	77.44	86.04	76.79	79.06	74.68
SVM	Unbalanced	76.65^*^	84.58^*^	79.42^*^	80.20^*^	78.71^*^
	Balanced	77.56	85.99	76.73	79.66	74.03
Adaboost	Unbalanced	76.58	84.53	79.58	79.50	79.72
	Balanced	77.18	85.85	76.59	78.62	74.68
KNN	Unbalanced	72.68	79.72	75.91	76.71	75.20
	Balanced	76.45	82.92	74.78	80.47	69.88
Random forest	Unbalanced	75.54	83.02	78.65	78.55	78.27
	Balanced	78.35^*^	86.89^*^	77.93^*^	82.86^*^	75.36^*^
Naïve Bayes	Unbalanced	73.90	81.85	75.09	82.83	68.71
	Balanced	75.75	82.64	73.97	79.90	68.91
Xgboost	Unbalanced	74.69	82.27	78.08	77.81	78.15
	Balanced	77.50	85.86	77.02	78.68	75.48

The asterisk sing (^*^) indicates the best performance

### Hyper parameter adjustment for Random Forest

For all models, including Random Forest, Scikit-learn offers a set of appropriate default hyper-parameters; however it might not necessarily yield the optimal outcome in all circumstances. To optimize the performance of random forests, a set of hyper parameters were changed with 100 trials on a particular search space using stratified 10-fold cross-validation. These variables had the following values: Max features is the number of features that each tree considers when splitting a node; min samples split is the smallest number of samples needed at a leaf node; n estimators is the total number of decision trees in the forest; and max samples is the total number of samples that must be taken from independent variables to train each tree. Table 4 shows both the optimized and default Scikit-learn hyper-parameters.

**Table 4:** Default and optimal tuned hyper parameters of Random Forest algorithm for predicting facility delivery and its determinants among reproductive-age women in Ethiopia (PMAET 2019, *n* = 5,413)

Hyper-parameters	Default	Optimal value
Number of tree	100	250
Features taken into account for the optimal split	square of the quantity of characteristics	0.85
minimal sample count needed to divide an internal node	2	6
minimum quantity of samples needed at a leaf node	1	2
The number of independent variable samples needed for training each base estimator	None	1

Ultimately, using balanced training data and adjusted hyper-parameters, a random forest model was built using 10-fold cross-validation. The model produced results including 79.51% accuracy, 86.78% areas under the curve, 80.06% precision, 75.60% recall, and 77.81% f1_score.

### Predicting health facility delivery

**Fig. 3:**
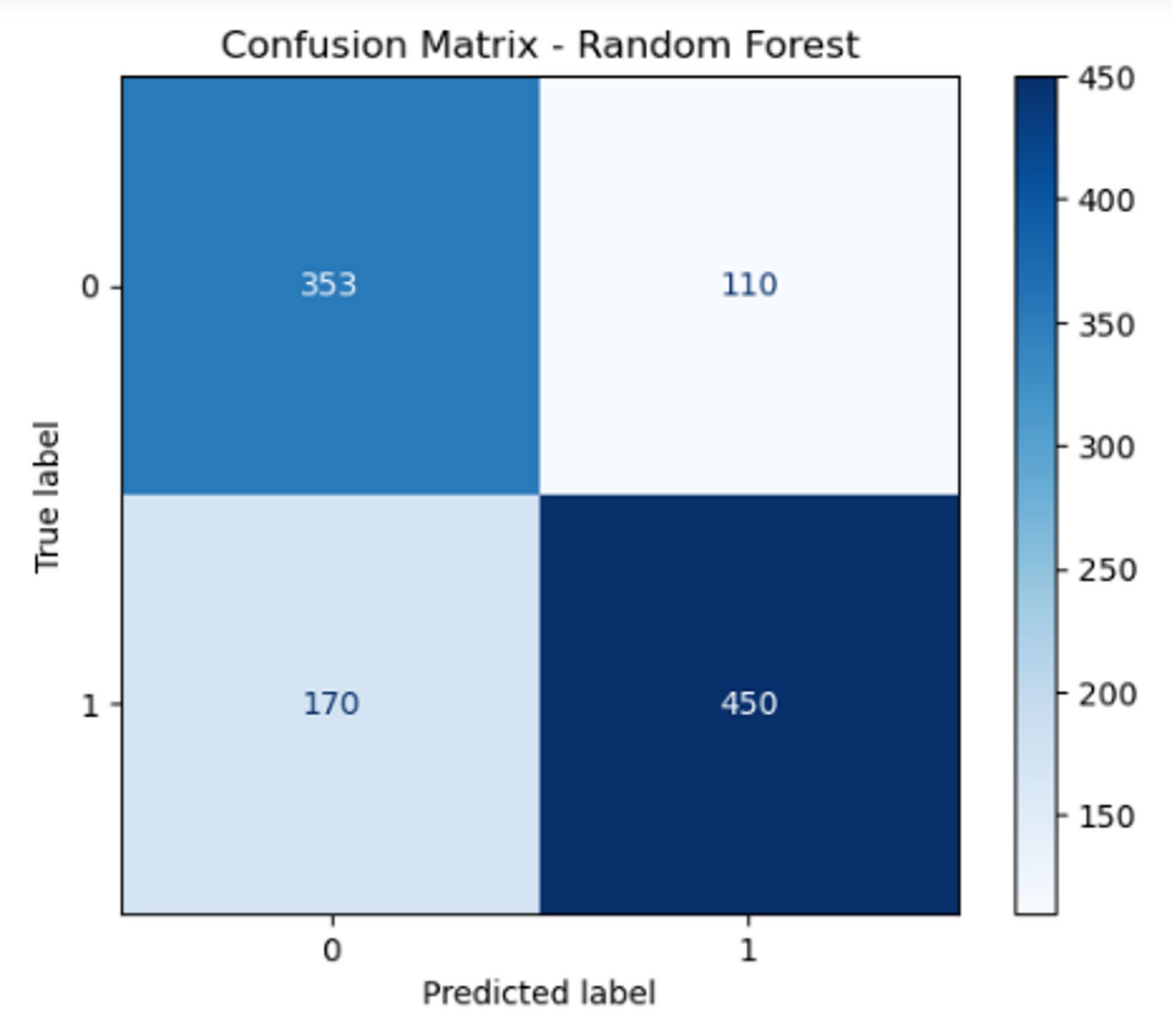
Confusion matrix of random forest model prediction on test dataset for predicting facility delivery and its determinants among reproductive-age women in Ethiopia (PMAET 2019, *n* = 5,413)

After training and improving the best model’s hyper parameters, 1083(20%) test samples were utilized to assess the random forest model’s performance. Out of 463 home delivery statuses, the model correctly predicted 353 (true negative). Out of 620 facility deliveries, the model predicted 450 as facility deliveries (a true positive). However, the model misclassified 110 home delivery statuses as facility deliveries (false positive) and 170 facility deliveries as home delivery statuses (false negative), as seen in (Fig. 3). Overall, the model predicted with 74.15% accuracy, 72.58% recall, 81.10% F1-score, and 80.36% precision on test data.

$$\begin{aligned}\mathrm{Accuracy}&\;=\;\mathrm{TP}+\;\mathrm{TN}/(\mathrm{TP}\;+\;\mathrm{FP}\;+\;\mathrm{FN}\;+\;\mathrm{TN})\\&\;=\;450\;+\;353/450\;+\;353,\;170\;+\;110\;=\;74.15\%\end{aligned}$$

$$\mathrm{Precision}\;=\;\mathrm{TP}/(\mathrm{TP}+\mathrm{FP})\;=\;450/450\;+\;110\;=\;80.36\%$$

$$\mathrm{Recall}\;=\;\mathrm{TP}/(\mathrm{TP}+\mathrm{FN})\;=\;450/450\;+\;170\;=\;72.58\%$$

The F1 score is calculated as (2 × Precision × Recall) / (Precision + Recall), which is 76.27%.

**Fig. 4:**
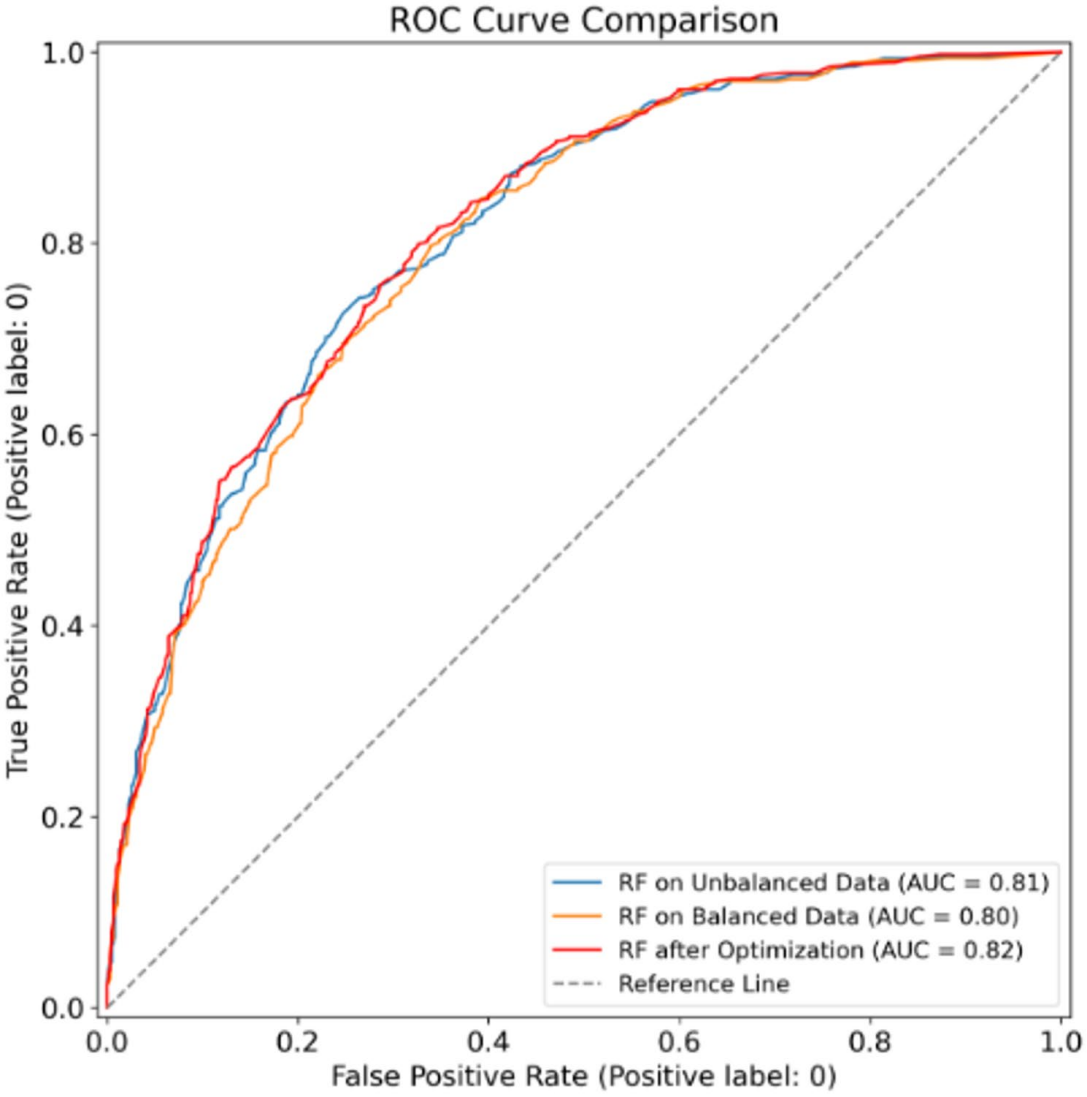
Comparison of Random Forest model’s prediction on test dataset for predicting facility delivery and its determinants among reproductive-age women in Ethiopia (PMAET 2019, *n* = 5,413)

Figure 4 shows the area under the receiver operating characteristic (ROC) curve comparison of the Random Forest model’s predictions on unbalanced and balanced data, as well as after model optimization. The ROC curve was used to summarize the model’s performance at different classification thresholds, showing how sensitivity and specificity change. This enables better understanding of the trade-offs between true and false positive rates, which aids in the setting of ideal thresholds to reduce misclassification errors. On unbalanced data, the Random Forest model achieved an AUC of 81%. After balancing and hyper parameter tweaking, the prediction on test data yielded AUCs of 80% and 82% respectively, suggesting a strong predictive model.

### Visualization of feature importance

**Fig. 5:**
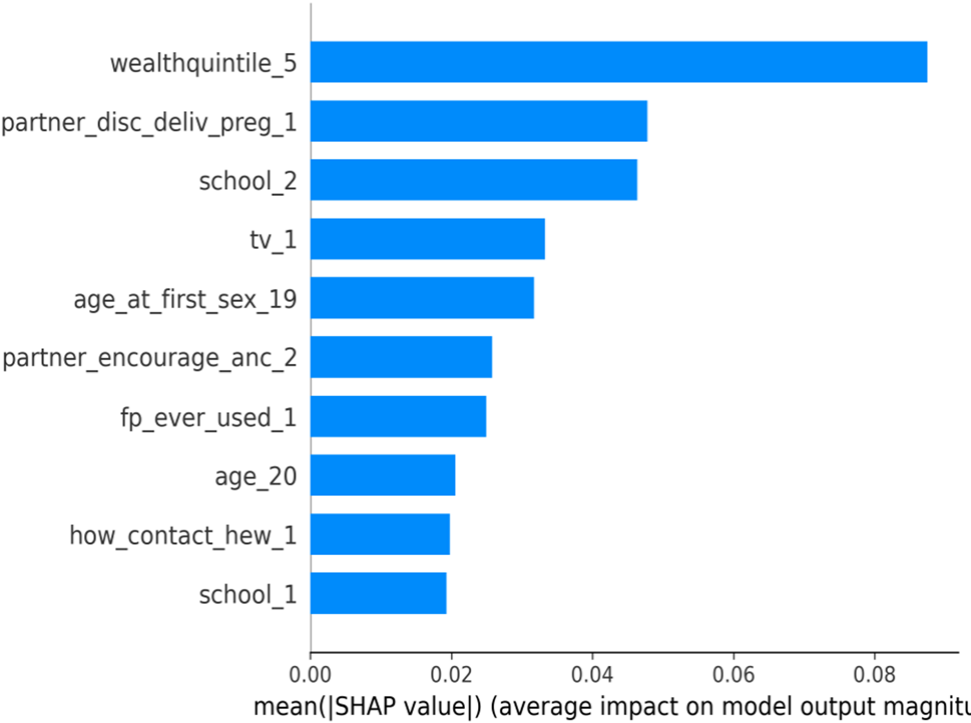
SHAP global feature importance plot of optimized Random Forest model for predicting facility delivery and its determinants among reproductive-age women in Ethiopia (PMAET 2019, *n* = 5,413)

Machine learning algorithms are made to adapt and learn from data without explicit rules, in contrast to classical statistical analysis, which is more organized and depends on pre-defined rules and formulas, such as employing p-value thresholds to identify significant features. Unfortunately, because it can be challenging to understand why ML models make particular predictions, they are frequently referred to as “black boxes”. This study selects the top predictors of health facility delivery using model-agnostic SHAP (SHapley Additive exPlanations) global feature importance in order to address this. This method quantifies the contribution of each characteristic to the anticipated facility delivery status by looking at the mean absolute SHAP value for each predictor across all the data. The SHAP global importance scores for the top 10 factors in the optimized random forest model are displayed in (Fig. 5). The attributes that exhibit greater mean absolute SHAP values are considered more significant, and the predictors are ranked in descending order of significance for the prediction of the outcome variable.

The results revealed that the most important factors to predict facility delivery are Highest wealth quintile (wealthquintile_5), secondary or above school attended (school_2), own TV (TV_1), partner did not encouraged ANC (partner_encourage_anc_2), discussed with partner where to deliver (partner_disc_deliv_preg_1), utilization of FP (fp_ever_used_1), Age at first sexual intercourse 19 years or above (age_at_first_sex_19), age between 20 and 34 (age_20), Know who to call for medical assistance if you go into labor(how_contsct_hew_1), and attended primary school (school_1).

### Model interpretation/explanation

**Fig. 6:**
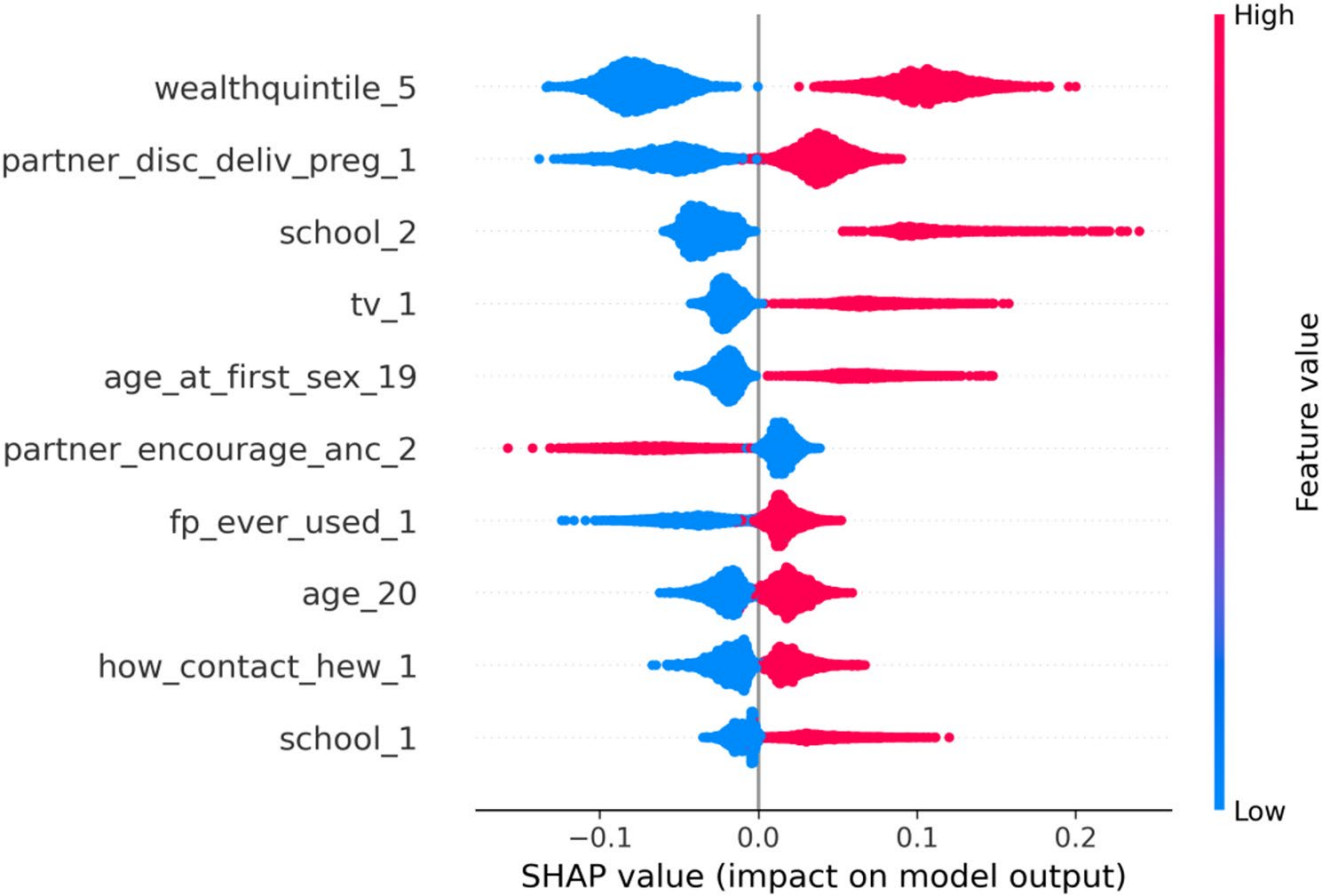
Beeswarm plot, ranked by mean absolute SHAP value generated by optimized Random Forest model for predicting facility delivery and its determinants among reproductive-age women in Ethiopia (PMAET 2019, *n* = 5,413)

A comprehensive image of how the elements affect the model’s overall predictions was provided using Beeswarms graph. Plotting the Shapley value of each individual predictor for each sample, (Fig. 6) shows the distribution of each predictor’s impact on the model’s output (i.e., facility delivery prediction).

Each of the top 10 characteristics’ significance and correlation with the outcome variable are shown by the points on this beeswarm plot, which depicts the Shapley values of the facility delivery status features. The figure’s red and blue tones correspond to each predictor variable’s higher and lower value.

The probability of facility delivery is higher at points that are right of the vertical line (0 SHAP value) and lower at those that are left of it. Given that each variable is categorical and has two possible values, category code 0 (low value) is represented by the blue line, and category code 1, or high value, is represented by the red line.

Features like highest wealth quintile (wealthquintile_5), education level secondary and above (school_2), discussed with partner where to deliver (partner_disc_deliv_preg_1), Age at first sexual intercourse 19 years and above (age_at_first_sex_19) and TV ownership (tv_1) mostly contribute positively to the prediction, meaning higher values for these features increase the likelihood of facility delivery. The Feature partner did not encouraged anc (partner_encourage_anc_2) predominantly shows negative impacts, indicating higher values for this feature decrease the likelihood of facility delivery. Finally the features such as using FP (fp_ever_used_1), age group 20–34 (age_20), and Knowing emergency contact if you go into labor(how_contsct_hew_1) show less impact, with a mix of positive and negative SHAP values.

**Fig. 7:**
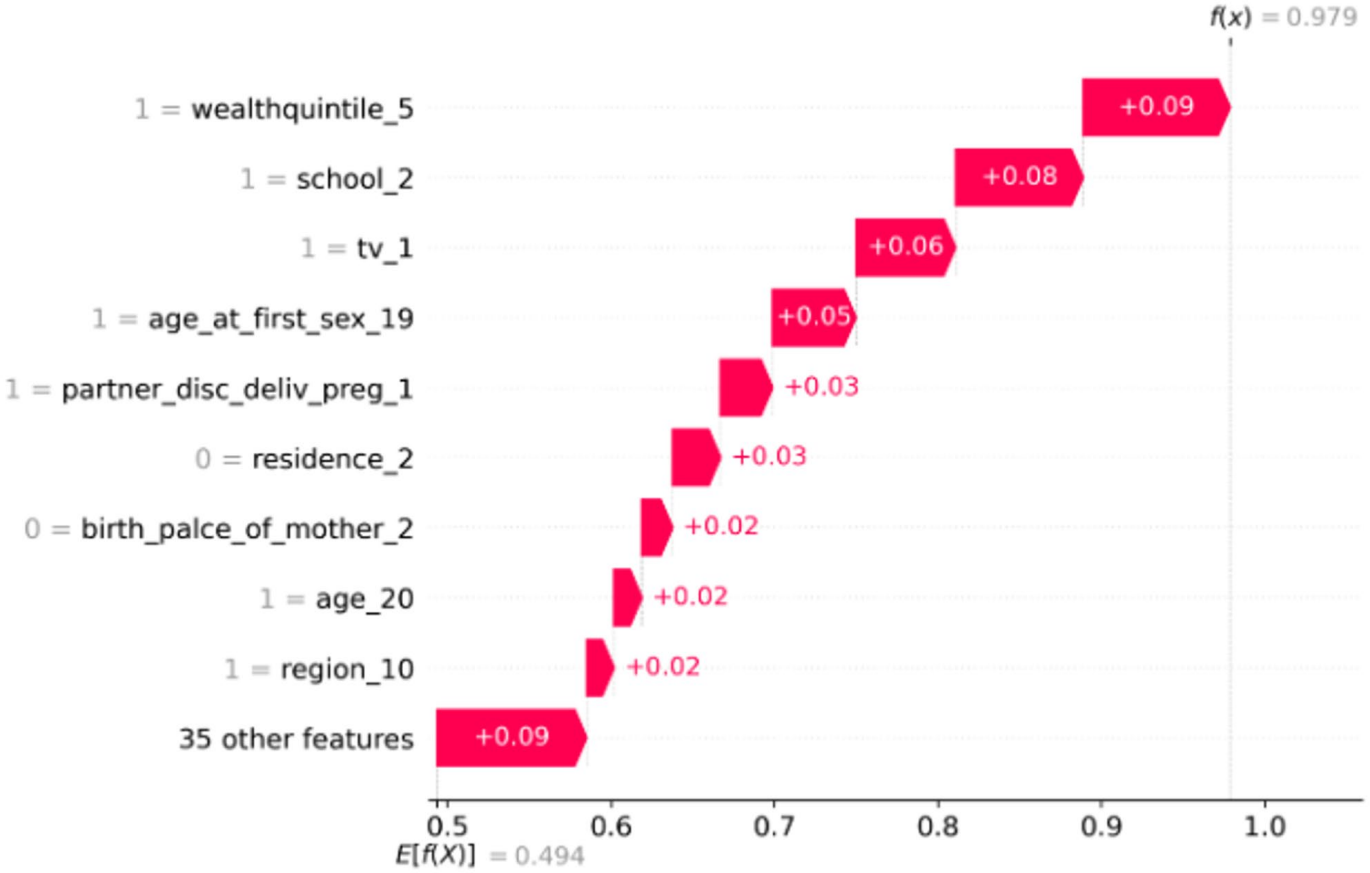
Waterfall plot, displaying prediction of second observation for predicting facility delivery and its determinants among reproductive-age women in Ethiopia (PMAET 2019, *n* = 5,413)

**Fig. 8:**
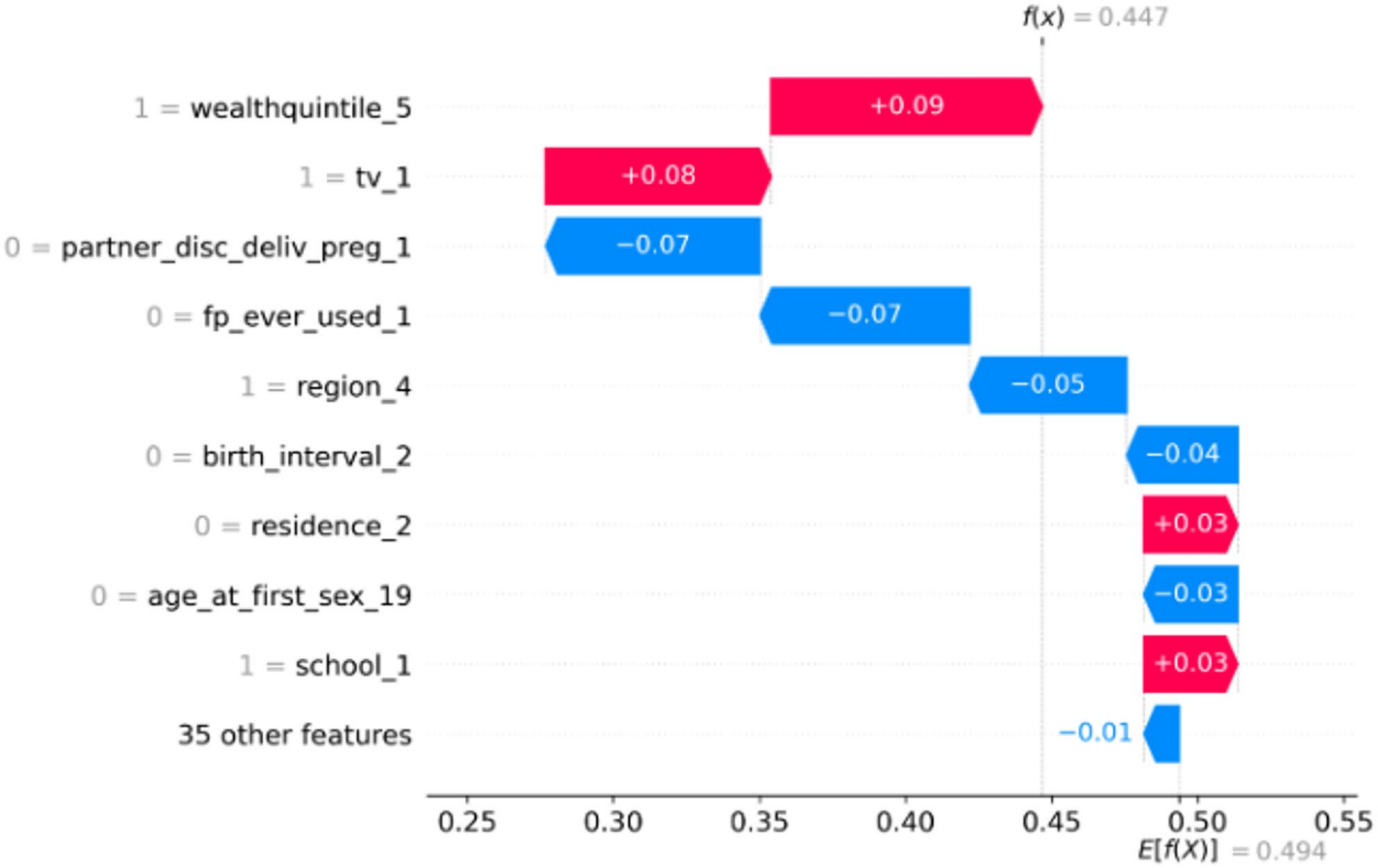
Waterfall plot, displaying prediction of the third observation for predicting facility delivery and its determinants among reproductive-age women in Ethiopia (PMAET 2019, *n* = 5,413)

At last, the model prediction of the second and third observations in (Figs. 7 and 8), respectively, was explained using waterfall plots (Local SHAP explanations). The waterfall plots in (Fig. 7) start with the expected value of the model output on the x-axis (E[f(X)] = 0.494), which is the first estimate for the sample before taking feature contributions into account. Usually, the average or most prevalent prediction for the dataset is this baseline prediction. 

When the model output for a specific observation is greater than this value (E [f(X)]), it indicates a positive class that is, “facility delivery” while scores below it indicate a negative class—that is, “home delivery.” As a result, for the second observation, the predicted value output is moved to the final model output (f(x) = 0.979), which is categorized as positive class (facility delivery), by the combination of the positive contributions (in red) and the negative contributions (in blue). Thus, in (Fig. 7) being in the highest wealth quintile (1=wealthquintile_5), attending secondary and above school(1=school_2), owning TV(1 = tv_1), being 19 years and above Age at first sexual intercourse (1= age_at_first_sex_19), discussed with partner where to deliver (1=partner_disc_deliv_preg_1), not being rural resident (0=residence_2), being age between 20–34(1=age_20), and being from Addis(1= region_10) drives up the probability of delivering at health facility.

Similar to the second observation, the third observation’s expected value output (E[f(x)] = 0.494) is moved to the final model output (f(x) = 0.447) by the sum of the positive (in red) and negative (in blue) contributions. This indicates that there is a (1.0–0.447) 55.3% chance that the observation will not be made in a health facility (negative class). Specifically for this woman being in the highest wealth quintile (1=wealthquintile_5), owning TV (1 = tv_1), attending primary school (1=school_2), and not being rural resident (0=residence_2) drives up the probability of delivering at health facility. While, not having years of birth interval (0= birth_interval_2), not being at the age of 19 years or above at first sexual intercourse (0= age_at_first_sex_19), not discussing with partner where to deliver (0=partner_disc_deliv_preg_1), not using family planning method (0 = fp_ever_used_1), and being from Oromia (1= region_4) drives down the probability of delivering at health facility, this is shown in (Fig. 8).

### Association rule mining

The analysis of the factors influencing facility delivery revealed several significant associations, which are supported by the findings from the association rule mining. The process of finding intriguing correlations between variables in big datasets is called association rule mining. In this study the association rule mining was conducted by utilizing Apriori algorithm in Python 3.11.5. The Apriori algorithm generated 30 facility delivery-related rules with a confidence level of over 85%, however only 9 rules with a confidence level of over 95% were produced, and three of them are presented as follow:-.

Rule I: IF “wealthquintile_5 = 1, tv_1 = 1, school_2 = 1” THEN the probability of fd_1 = true (confidence = 0.951) This rule suggests that individuals who: Belong to the highest wealth quintile (wealthquintile_5 = 1), Have access to television (tv_1 = 1), Have attended secondary and above school (school_2 = 1), is highly likely to choose facility delivery (fd_1), with 95.1% confidence, indicates that women in the highest wealth quintile, owning a TV, and having secondary or higher education have a very high probability of delivering in a health facility.

Rule II: IF “partner_encourage_anc_2 = 0, school_2 = 1, tv_1 = 1” THEN the probability of fd_1 = true (confidence = 0.952) This rule suggests that individuals who: Have a partner who encourages antenatal care (partner_encourage_anc_2 = 0), Have attended secondary and above school (school_2 = 1), owns television (tv_1 = 1) are highly likely to choose facility delivery (fd_1), with confidence of 95.2%, demonstrates the impact of partner support combined with educational attainment and media exposure on facility delivery decisions.

Rule III: IF “fp_ever_used_1 = 0, partner_encourage_anc_2 = 1, school_2 = 1” THEN the probability of fd_1 = true (confidence = 0.864): This rule suggests that individuals who: Have never used family planning methods (fp_ever_used_1 = 0), Have a partner who does not encourage antenatal care (partner_encourage_anc_2 = 1), Have attended secondary and above school (school_2 = 1) are likely to choose facility delivery (fd_1), with 86.4% confidence, indicates that even when family planning is not used and there is no partner encouragement, being secondary and above level of education still plays a significant role in ensuring facility delivery.

## Discussion

This study aimed to predict facility delivery and its determinants among Ethiopian women of reproductive age using machine learning classifiers. By employing techniques like tenfold cross-validation on both balanced and imbalanced data, the study compared the performance of seven classifiers using metrics such as accuracy, F1 score, precision, recall, and AUC score. Machine learning models offered significant advantages over traditional methods, including the ability to handle high-dimensional data, capture non-linear relationships, and provide robust performance metrics.

The best model identified in this study for predicting facility delivery was Random Forest, with 78.35% of accuracy, 86.89% areas under the ROC curve, 82.86% precision, 75.36% recall, and 77.93%F1_score based on tenfold cross validation score on balanced training data. This model’s selection aligns with research in Ethiopia on Prediction of contraceptive discontinuation among reproductive-age women in Ethiopia using Ethiopian Demographic and Health Survey 2016 Dataset [51], where Random Forest has demonstrated superior performance. However, the performance was significantly lower than that of another study conducted in Ethiopian [52], which found J48 to be the best model for predicting skilled delivery services in Ethiopia, with an accuracy of (98%), sensitivity (96%), specificity (99%), and area under the ROC (98%). These inconsistencies could be attributed to the size of the dataset used in model development, because the previous study included 11,023 records, allowing the model to learn well and forecast with greater accuracy.

Finally, 1,083 (20%) test samples were used to predict facility delivery after training and optimizing the hyper parameters of the best model, Random Forest. The model accurately predicted 450 facility deliveries but misclassified 170 as home deliveries out of 620 actual facility deliveries. With 74.15% accuracy, 72.58% recall, 81.10% F1-score, and 80.36% of precision on the test data, the Random Forest model was the best predictive model in this study.

The SHAP analysis based on the RF model revealed that Highest wealth quintile, school level secondary and above, TV ownership, partner who encouraged ANC, discussion with partner where to deliver, using family planning method, Age at first sexual intercourse 19 years or above, age between 20 and 34 years old, Knowledge of emergency contact if you go into labor, and primary school were the top ten important predictors of facility delivery in Ethiopia. Finally, SHAP model explanation was applied to interpret how each predictor affects the facility delivery.

The SHAP model explanation states that women in the highest wealth quintile are more likely to give birth in a health facility. This highlights the significant impact of socio-economic status on health facility delivery. This finding aligns with the results of a population-based cross-sectional study conducted in Ethiopia [20], which found that women with rich wealth quintile were more likely to give birth at a health facility. Additional researches conducted in Ethiopia also revealed that women in richest wealth quintile category had increased likelihood of health facility delivery [34, 53] likewise this finding was consistent with study conducted in Nigeria [54]. A possible explanation could be that mothers from low-income households are unable to afford indirect expenses such as transportation. In addition, mothers from poorer socioeconomic backgrounds may face barriers in accessing health information. According to this research, increased use of institutional delivery services is highly dependent on improving economic condition.

Women with a secondary or higher education level are more likely to deliver at health facilities. This research emphasizes the importance of educational achievement, since educated women are more likely to recognize the benefits of delivery in health facilities. A study conducted in Ethiopia supports this finding, revealing a higher likelihood of facility delivery among women with secondary and higher education levels [19].also another study conducted in Togo reported that Mothers who have completed secondary education or higher are more likely to use facility delivery services [55]. This may be educated mothers are more aware of the benefits of skilled birth attendance and institutional deliveries, and they are better positioned to make informed health decisions.

There is a high probability of a facility delivery when a woman and her husband discuss the delivery location. This is consistent with a study that was done in a pastoral village in the Dubti district of Afar, northeastern Ethiopia, and found that women whose husbands were involved in selecting the delivery location were more likely to give birth in a medical facility [56]. Furthermore, the results of a different study carried out in Bangladesh, Nepal, and Pakistan, three South Asian nations, are consistent with it [57], Women are more likely to give birth in medical facilities if they talk to their partners about their delivery plans. Possibly this could be spousal communication and involvement in delivery decisions provides emotional support, practical assistance, and confidence in accessing healthcare services, while also raising awareness about the benefits of facility-based deliveries and the need for preparedness, ultimately increasing the likelihood of women giving birth in medical facilities.

Media Exposure (TV Ownership) was yet another extremely strong predictor of facility delivery. Those women who own TV have higher probability of facility delivery, numerous cross-sectional investigations carried out throughout Ethiopia provided evidence in support of this finding reporting that Ownership of a television and exposure to health messages through media significantly enhance the likelihood of facility delivery [20, 31, 34, 53]. Also one more study from Madagascar supports this finding [58].This is likely due to Media serves as a critical channel for disseminating health information and encouraging health-seeking behaviors.

Furthermore, this study discovered that partner encouragement for ANC enhances the possibility of facility delivery. This is consistent with a qualitative study conducted in Sidama zone, Ethiopia, which found that women encouraged by their partners to attend ANC were more likely to use facility delivery services [59]. This might be to begin; partner support frequently translates into emotional, financial, and logistical assistance, making it easier for women to access and use health care. When partners are actively involved, they can help overcome obstacles such as healthcare expenditures and time restraints. Second, partner encouragement can improve a woman’s health-seeking behavior by emphasizing the value of professional treatment during pregnancy and childbirth.

Also this study revealed that another factor influencing facility delivery is the use of family planning methods. When practicing family planning, women are more likely to give birth in medical facilities. This finding is consistent with a qualitative research conducted in Ethiopia, which found that integrating family planning with basic healthcare services enhanced service delivery outcomes, including higher adoption of facility-based deliveries due to increased involvement with healthcare services [60]. Also in line with a study conducted in sub-Saharan Africa [61], which highlighted that women who used modern contraceptive methods were more likely to deliver in a health facility. The use of family planning is a sign of a woman’s involvement with the medical system and this is frequently associated with greater rates of institutional delivery. This finding can be attributed to several reasons: increased interaction with the healthcare system enhances their awareness and familiarity with medical services; health education associated with family planning informs them of the benefits of professional childbirth care; empowerment and autonomy gained through family planning lead to informed delivery choices; continuity of care from regular healthcare provider contact ensures timely access to antenatal care; and reduced fear and misconceptions about facility delivery arise from regular interactions with healthcare professionals.

The age of women is also one of the top ten predictors of facility delivery. Women aged 20 to 34 are more likely to give birth in a health facility. The bulk of studies conducted in Ethiopia and other countries found a significant link between facility delivery and age between 20 and 34 [19, 28, 56, 61]. Similarly Research analyzing data from the Nigeria Demographic and Health Survey (NDHS) indicated that maternal age is a critical factor for facility delivery. The study showed that women aged 20–34 are more likely to deliver in health facilities compared to younger and older age groups [36]. This might be due to factors such as increased maturity, financial stability, and health awareness. The consistency across multiple studies in different contexts underscores the importance of maternal age as a key determinant of facility delivery.

Another top predictor of facility delivery was age of women at first sexual intercourse. Women aged 19 and older have a higher probability of delivering their child in a healthcare facility. This finding was supported by study conducted in Ethiopia reported that later age at first sexual intercourse was associated with institutional delivery [16]. Possible explanations for this finding include increased maturity and decision-making capacity, a higher likelihood of being in stable and supportive relationships, and improved access to health education and resources, all of which promote the use of healthcare facilities for childbirth.

Finally, the mother’s knowledge of emergency contact was among the top ten predictors of facility delivery. Women who Know who to call for medical assistance if they go into labor have an increased likelihood of facility deliver, and this finding is supported by studies conducted in Ethiopia which emphasized that women who are knowledgeable about emergency contact numbers for medical assistance during labor are more likely to utilize facility-based delivery services [53, 62]. Additionally, a comprehensive review of facility-based delivery in low- and middle-income countries identified the importance of emergency preparedness, including knowing who to contact, as a key facilitator for institutional deliveries [63]. This could be because knowing of emergency contact allows women and their families to respond quickly during labor. When women know who to call for quick medical assistance, such as an ambulance service or their local health institution, they are more prepared and confident in getting appropriate care. 

### Strengths and limitations

#### Strengths

This study used supervised machine learning approaches, such as SHAP (Shapley Additive Explanations), to examine a vast and comprehensive dataset, increasing the reliability and validity of the results. The SHAP analysis offers information about the relative importance and contribution of individual features to the model’s predictions. Furthermore, SHAP’s capacity to read black-box models provides a deep understanding of how each characteristic influences individual predictions, improving the interpretability of the model’s results. Using SHAP for both global and local feature importance aids in describing complex model behaviors, allowing users to better understand and trust the model’s judgments. This comprehensive approach ensures a sophisticated grasp of the elements driving health facility delivery.

#### Limitations

There are a number of limitations to this study that should be taken into account when interpreting the results. First, the dataset’s cross-sectional design makes it more difficult to determine the causal linkages between health facility delivery and variables. Second, because the analysis was based on secondary data from the PMA Ethiopia 2019 survey, the study was limited to the variables found in the dataset. As a result, the predicted models were unable to incorporate certain significant factors mentioned in earlier research, such as trip duration, distance to medical facilities, and transportation accessibility.

Furthermore, since the data were gathered in 2019, the COVID-19 pandemic and other contextual changes may have altered the patterns of maternal health service consumption. Additionally, there was no external validation using a different dataset, which could restrict how broadly the results can be applied. Lastly, some variables may be prone to recall or social desirability bias because they were based on self-reported replies from survey respondents.

## Conclusion

This research employed machine learning techniques to ascertain the primary factors influencing health facility deliveries among women of reproductive age in Ethiopia, utilizing nationally representative survey data. The results underscore the significant impact of mother education, household affluence, antenatal care engagement, parity, and geographic location on facility-based delivery rates. The application of interpretable machine learning methods, such as SHAP analysis, yielded enhanced understanding of the relative significance and influence of these predictors.

The findings highlight the need for focused interventions that enhance women’s education, increase access to prenatal care services, and address socioeconomic and regional inequities in the use of maternal health services from a policy and programmatic standpoint. In terms of methodology, this work shows how useful it is to assist evidence-based decision-making in maternal and public health by integrating explainability methodologies with predictive machine learning models. In order to further improve maternal health measures, future research may build on this work by using longitudinal data and investigating causal pathways.

## Data Availability

The datasets analyzed during the current study are publicly available from the Performance Monitoring for Action (PMA) data repository upon reasonable request and registration through the official PMA data access portal: PMA Available Datasets.

## Abbreviations

AdaBoost: Adaptive Boosting
EA: Enumeration Area
FN: False Negative
FP: False positive
HEW: Health Extension Worker
KNN: K-Nearest Neighbor
ML: Machine Learning
MLM: Machine Learning Model
MPH: Master of Public Health
MMR: Maternal Mortality Rate
PMA: Performance Monitoring for Action
RFECV: Recursive Feature Elimination with Cross Validation
ROC: Receiver Operating Characteristic
SMOTE: Synthetic Minority Oversampling Technique
SVM: Support Vector Machine
TN: True Negative
TP: True Positive
WHO: World Health Organization
XGBoost: eXtreme Gradient Boosting
